# Practical Medicine

**Published:** 1878-04

**Authors:** 


### Practical Medicine.

A Case of Thrombosis of one Coronary Artery. Dr. H. Hammer, of St. Louis. *Wiener Med. Wochenschrift*, Feb. 2, 1878).

The patient, a robust man of 34 years, had had various attacks of rheumatism since a year, and was at the time recovering from a severe attack. No valvular lesion existed. While he was sitting in a chair, collapse set in suddenly at one o'clock at noon. Called in consultation Dr. H., found him the next morning in collapse, but with undisturbed mind, pallor, cyanotic coloration of the skin and clammy sweat but *no* dyspnœa, cough or pain. Respirations 24 and pulse eight beats per minute. Percussion of lungs and heart and auscultation of lungs essentially negative. On listening to the cardiac sounds, the feeble but pure systolic and diastolic sounds were heard; each cardiac contraction lasting about a second, was followed by a vibrating sound of five seconds duration, referable to clonic spasm of the heart; after two seconds of absolute rest the cycle was repeated.

The sudden collapse, the cardiac symptoms and the absence of other causes induced Dr. Hammer to state the anti-mortem diagnosis as occlusion of one coronary artery—the first diagnosis of its kind. Death occurred after 19 hours.

The heart was found normal; of the valves, one, the posterior fold of the aortic semilunar valve, was altered. There were adhesions between it and the wall of the aorta and it was surrounded with endocarditic excrescences from which a clot extended into the right sinus valsavæ and thence down into the coronary artery.

